# Clinical Remarks upon Surgical Cases in the Buffalo General Hospital—Excision of Two-thirds of the Humerus in a Child Two and a Half Years of Age—Varicose Veins and Ulcers—Lumbar Abscess

**Published:** 1866-01

**Authors:** J. F. Miner


ART. IV.—*Clinical Remarks upon Surgical Cases in the Buffalo General Hospital—Excision of two-thirds of the Humerus in a Child two and a half years of age—Varicose Veins and Ulcers—Lumbar Abscess.* By J. F. MINER, M. D.

GENTLEMEN—I desire in the first place to show the results of an operation which you have previously witnessed:

PATRICK K—— is the man from whose shoulder we removed the head of the humerus about six weeks since; he has come up from his home desirous of showing what the operation has done for him. As you will recollect, he had suffered for two years from pain in the joint and discharge in such degree as to induce a condition of great debility, giving him the appearance of a patient in the last stage of some fatal malady. Since the operation he has suffered nothing of his former pain, the suppuration soon ceased, and the wound healed entirely. His appetite and strength have returned, and his friends can scarcely believe he is the same man. The removal, then, of the carious head of the humerus is shown, in this instance at least, to have been a very judicious and well-advised procedure; by it our patient has been immediately restored to comparative soundness. You may observe by the way he moves his arm that most of the functions are retained; he can use it perfectly in some respects, while in others he cannot; cannot raise it with much force at right angles with the body, but it is quite strong in all other directions. There is a new connection made with the glenoid cavity, ligamentous in character, which answers to fix in some degree the upper end of the humerus, but of course it is loosely fixed and consequently not a mechanical improvement upon the original plan of making this joint, but it must be acknowledged a very good imitation, and worth vastly more than could have been expected.

*Excision of the Humerus.*—The little child which we now bring before you, fully under the influence of sulphuric ether, is about to undergo the same or a similar operation as that made upon the man who is so proud of his restored arm. The little child when very young—six or eight months of age—commenced to suffer from redness, tumefaction, and very great pain on motion of the arm. Abscesses formed and were opened, and very soon it was evident that somewhere the bones constituting the shoulder-joint were diseased. The little child fell under my observation through the kindness of my friend Dr. Nichell, the nature of the difficulty being at the time perfectly obvious. It was very feeble during that season, looking as if it would receive but few attentions more in this life. While the child was under the care of Dr. Nichell we promised the mother that when it had gained flesh and strength and the warm season had passed, we would make operation for removal of dead bone. It appears remarkable that the child should have recovered from the condition of debility, but so it is, and now is apparently well nourished; the local disease however is the same, or certainly, no better. The discharge of pus from the various openings around the joint and over the body of the bone, is as profuse as possible, while a probe passes down in many places upon dead bone. Dr. Otto Berger, who has recently had charge of the patient, has accepted the offer made the mother so long since, and kindly presented his patient before you for the removal of whatever of dead bone may be found. In the usual way we have opened by a single straight incision down upon the head of the humerus, which is found completely carious; caries in bone, answering as you will recollect to ulceration in the soft parts. This condition of disease extends down the body of the bone, and the periostium is very easily separated from its attachment. The bone is divided carefully and smoothly with the saw, and two-thirds of the whole bone removed.

This looks like a severe operation for our little patient, but it would be difficult to conceive how removal of such a diseased structure could be worse than letting it alone. If we are successful in our operation and correct in our expectations we shall achieve a real success, the little child will recover and be freed from this terrible drain upon the constitution and life. The arm will not be useful and strong like the other, but as you have just seen, nature is competent to provide a new attachment of the bone—a ligamatous extension will grow from the divided bone, and remaining periosteum, and the little child will have a useful hand, worth a thousand times more than an artificial one; but of course the operation is not unattended by danger, and our little patient may disappoint us altogether. Such an operation now takes the place of what would formerly have been amputation at the shoulder joint. Its advantages arise from two facts; it is less dangerous to the life of the patient, and if successful the arm looks better and is much more useful than any artificial one, even though we have been obliged to remove so much of the bone. This is conservative surgery; avoiding mutilation and still removing the disease; it is a case eminently illustrative of true conservatism. You have heard of conservative surgery, a term used by many to apologize for the most stupid and unjustifiable inactivity, but it should be used in a widely different sense. Conservative surgery means active, earnest, well-advised, operative interference in all cases where positive benefits may be gained—where organs and functions may be preserved or restored. Of our success in this case of conservatism we will inform you when results are determined.

*Varicose Veins.*—We have another case similar to the one I described to you and operated upon a few days since. The results in the other case were satisfactory, and it has not fallen to me to know of a single case which was treated skillfully by this method of injecting solution of persulphate of iron, but has proved satisfactory; and I have tested it by numerous trials. We then repeat the same operation—inject three drops of the solution diluted with thirty of water into the dilated vein; this is done in two or three places where the vessel is large or has enlarged branches. The ulcers will disappear after the veins are obliterated by a process I have before explained.

*Lumbar Abscess.*—Mr. C——, aged 48, of rather delicate appearance, has been this morning admitted from the medical to the surgical ward, and presents for treatment a tumefaction in the lumbar region of not more than four or five weeks' standing. It is tender upon pressure of the spinal bones just above the swelling; he walks in a stooping and very careful manner, and complains of great pain in the back. Upon examination there is evident fluctuation, and from the history, appearance and symptoms there is no doubt our patient is suffering from what is called lumbar abscess. In consequence of disease of the vertibræ pus often forms at the lateral aspect of the spine, and when thus appearing it has been called lumbar abscess. If it follows the proas muscle and appears in front it has been called proas abscess, but wherever it may appear it is disease of the vertabræ, and should be so regarded and treated. This form of disease has also been regarded as a strumous disease and liable only to arise in persons of a strumous constitution. More recently other opinions have been urged, and the view entertained that injuries may cause it even in healthy constitutions—that it more generally arises from traumatic causes. This patient is brought before you early in the disease which is quite remarkable, most cases not coming under the observation of the surgeon, until much farther advanced. The fluctuation is not very distinct, and the pus is as yet deep beneath the muscles of the lumbar region. The pain is severe, relish for food gone, sleep is disturbed, chills followed by perspiration are frequently repeated, the system generally is disturbed and the patient has a worn, anxious expression, and is impatient of relief from pain.

Clinical experience has led to some differences of opinion as to the proper management of such abscess. Some surgeons advising that it be left unopened, and others claiming that to allow the escape of the pus is necessary for the relief of pain and is unproductive of injury. In the very early stage in which we find this disease in this instance, it is believed that evacuation of the pus may relieve pain and possibly be of service in other respects. I have determined in this case to puncture and verify my diagnosis, and if possible relieve the constant and depressing pain our patient suffers, believing that pus as an almost universal rule, may better be allowed an escape than to be retained, and hoping that in this early stage of the disease some permanent improvement may be gained. Differences of opinion even in this case are entertained, and I have been advised by distinguished practitioners in this department not to interfere, but allow nature to pursue its own course. I had promised my patient relief by evacuation, and had made up my mind to that course. I am hardly now in condition to change without some new light or new fact, and prefer to try the plan I had proposed. Later in the disease it is not to be doubted, that whatever course may be adopted the result is about the same, sooner or later the malady is fatal. The proposition to evacuate sub-cutaneously, by valvular incision, or with exhausting syringe, to avoid admission of air, is probably, not of the slightest importance. The operation in advanced disease, in whatever way performed, is often followed by hectic fever, rapid failure of the health and strength and early fatal termination, and there is reason for the belief that in such cases life is longer continued without surgical interference.

